# Covid19 pandemic impacts on mental health of tunisian health care workers

**DOI:** 10.1192/j.eurpsy.2021.771

**Published:** 2021-08-13

**Authors:** M. Dhemaid, W. Abbes, A. Kerkeni, S. Bader, M. Abbes, K. Medhaffer, K. Zitoun, L. Ghanmi

**Affiliations:** Psychiatry, regional hospital of Gabes, Gabes, Tunisia

**Keywords:** health care workers, mental health, COVID-19 pandemic, anxiety depression

## Abstract

**Introduction:**

Covid19 pandemic in Tunisia has disturbed the health system. Health care workers, who were in the frontline to face this disease, had experienced reactions of anxiety, depression and distress.

**Objectives:**

To assess the level of anxiety and depression among health care workers of regional hospital of Gabes (south of Tunisia) and its associated factors.

**Methods:**

We conducted a cross-sectional study, from April 19, 2020, to May 5, 2020 on healthcare workers in Gabes regional Hospital. All hospital departments and units were included. We used a self-administered anonymous questionnaire containing sociodemographic and clinical data. Hospital Anxiety and Depression Scale (HAD) validated in the Tunisian dialectal version was used to assess anxiety and depression.

**Results:**

Among the 289 responding participants, 100 (34.6%) were frontline health care workers directly engaged in managing patients with coronavirus disease. Our study revealed that 43.6% of health care workers were suffering from anxiety and 44.3 % from depression. There was a significant association between anxiety and female gender (p<0.001), being married (p=0.006), mental health conditions (p<0.001) especially anxiety disorders (p<0.001) and depressive disorders (p=0.03), personal medical history of dysthyroidism (p=0.013) and smoking (p=0.005). Nurses experienced more likely anxiety symptoms than other occupation (p=0.021). There was significant relationship between depression and female gender (p<0.001), married status (p=0.02), age under 50 (p=0.032) and mental health conditions (p<0.001) such as anxiety disorders (p=0.001) and depressive disorders (p=0.013).

**Conclusions:**

It is crucial to provide care for health care workers with mental health problems during their struggle with covid19.

